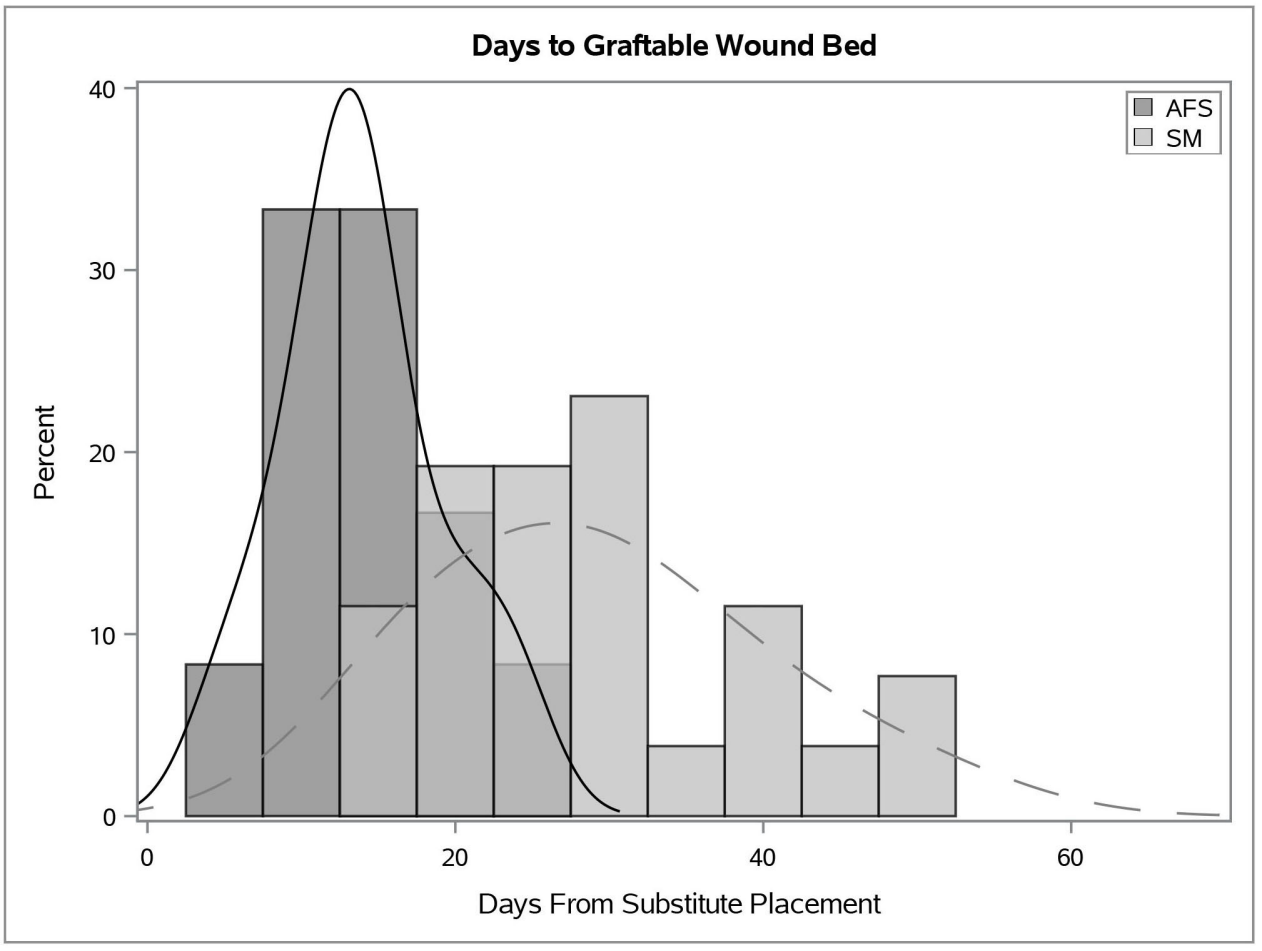# 552 Retrospective, Cohort Study Evaluating Time to Graft Between Acellular Fish Skin and Synthetic Matrix

**DOI:** 10.1093/jbcr/iraf019.181

**Published:** 2025-04-01

**Authors:** Mahmoud Hassouba, Muntazim Mukit, Kais Atmeh, Karapet Davtyan, Dani Kruchevsky, Ram Velamuri, Xiangxia Liu, David Hill

**Affiliations:** University of Tennessee Health Science Center; University of Tennessee Health Science Center; University of Tennessee Health Science Center; University of Tennessee Health Science Center; University of Tennessee Health Science Center; University of South Florida Morsani College of Medicine; University of Tennessee Health Science Center; Regional One Health

## Abstract

**Introduction:**

An estimated 23 million emergency department visits in the United States each year are due to traumatic wounds, accounting for trillions of medical costs and work and quality of life loss. The goal is to close the wound as quickly as is feasible, while managing the many complexities involved in the patients’ care. We hypothesized the use of acellular fish skin (AFS) would result in faster time to obtain a graftable wound bed compared to synthetic matrix (SM).

**Methods:**

This retrospective analysis was conducted for admission between July 2019 and April 2024. Patients receiving AFS for full thickness acute traumatic wounds were matched 1:3 by age and wound type to a group treated using SM. According to power analysis to find a minimum 10-day difference between the two treatments, we planned to enroll at least 32 patients. Two surgeons reviewed electronic charts for primary outcome. Incomplete charts were excluded, particularly when pictures or documentation were not available to assess the primary outcome. SAS 9.4 was used for analysis. Regression was utilized to adjust findings to differences in groups using manual backwards elimination.

**Results:**

Data was collected on 80 patients. Forty-two patients were excluded due to inability to adequately assess the primary outcome or for lack of at least 1 paired comparator. The final cohort included 38 patients [AFS (n = 12) and SM (n = 26)]. The mean age was 42.4 ± 20.7 years with 60.5% being male and equal proportions having burns treated vs another traumatic wound. The most common site was lower extremity. The only differences between groups were total body surface area (TBSA) burn percent, wound size, and use of negative pressure wound therapy (NPWT). All AFS patients had NPWT placed at time of implantation, vs. 50% of SM (p=0.001). The SM group had a larger TBSA (p< 0.001) and wound size (p=0.001), which were subsequently used to adjust primary outcome via regression. Patients with AFS had a faster time to graftable wound bed compared to SM (mean ± SD: 13.9 ± 5.3 vs 29.1 ± 10.3, p< 0.001).

**Conclusions:**

This study highlights that readiness for grafting is significantly improved when using AFS as a skin substitute for full-thickness traumatic injuries, compared to a synthetic matrix, leading to faster grafting readiness and wound closure.

**Applicability of Research to Practice:**

Evaluate and compare different skin substitutes

**Funding for the Study:**

N/A